# HBV Transmission Knowledge Among Korean-American Chronic Hepatitis B Patients in the United States

**DOI:** 10.1007/s10900-024-01412-y

**Published:** 2024-11-02

**Authors:** Giyoung Lee, HeeSoon Juon, Eunji Kim, Katherine C. Smith, Hie-Won Hann, Mimi Chang, Ann C. Klassen

**Affiliations:** 1 Department of Community Health and Prevention, Drexel University Dornsife School of Public Health, 3215 Market Street, 4th Floor, Philadelphia, PA 19104 USA; 2 Department of Medical Oncology, Thomas Jefferson University, Philadelphia, PA USA; 3 Department of Health, Behavior and Society, Johns Hopkins Bloomberg School of Public Health, Baltimore, MD USA; 4 Division of Gastroenterology and Hepatology, Thomas Jefferson Hospital, Philadelphia, PA USA; 5 Asian Pacific Liver Center, Coalition of Inclusive Medicine, Los Angeles, CA USA

**Keywords:** Hepatitis B, Asian/Pacific Islanders, Knowledge, Prevention, Transmission

## Abstract

Chronic hepatitis B (CHB) is a condition that disproportionately affects Asian Americans in the United States. Knowledge of transmission is crucial for CHB patients to practice prevention methods to limit the spread of the hepatitis B virus (HBV), but also live their lives free from unwarranted fears or restrictions. Among Asian CHB patients, several misperceptions about HBV transmission have been identified. This analysis aims to assess the current state of HBV knowledge among a cohort of Korean-American CHB patients. This mixed-methods study includes 363 respondents who completed a survey in either Korean (*N* = 298) or English (*N* = 65) at two clinical care settings in Philadelphia (*N* = 161) and Los Angeles (*N* = 202); 30 participants also completed in-depth interviews. Knowledge was measured on a 10-point scale, asking patients yes or no transmission questions (*n* = 10, alpha = 0.87). The average knowledge score was 6.3. In multivariate analyses, older age was associated with lower knowledge (β=-0.25, *p* < 0.001). More years of formal education (β = 0.09, *p* = 0.076) and utilizing more sources for health information (β = 0.12, *p* = 0.023) were both independently associated with higher knowledge scores. Qualitative findings show that misperceptions about transmission through shared food still exist and that provider communication is an important part of knowledge acquisition. These results suggest that despite receiving specialized, culturally concordant medical care for their disease, some Korean-American CHB patients have an inadequate understanding of transmission and that opportunities exist to improve education in this population. Identifying additional factors that influence knowledge acquisition and retention is key to developing culturally effective education interventions for this population.

## Introduction

Chronic hepatitis B (CHB) is a condition that disproportionately affects Asian Americans in the United States (US). Although Asian Americans only make up 6% of the US population, this group makes up approximately 58% of all hepatitis B cases [1]. Many of these cases can be attributed to immigrants arriving from Asian HBV-endemic countries [1]. In the US, hepatitis B virus (HBV) screening rates among Asian Americans have previously been found to be low (< 50%) [2]. Delays in treatment from undiagnosed HBV can lead to hepatocellular carcinoma and other hepatitis B-related morbidity and mortality [3]. Knowledge and awareness of HBV are associated with better healthcare-seeking behaviors [4, 5]. Sociodemographic factors associated with better knowledge include younger age, higher English proficiency, education attainment, and socioeconomic status [4, 6, 7].

Even after diagnosis, having knowledge of transmission is crucial for patients to continue practicing prevention strategies to limit the spread of HBV. The most common route of transmission for HBV is through perinatal transmission, sexual intercourse, and contact with infected blood [8]. Among Asian CHB patients, several misperceptions about HBV transmission have been identified, such as spreading HBV through the sharing of food, eating and drinking utensils, and breastfeeding [6, 9, 10]. These misperceptions can lead to increased social distancing and stigma against people with CHB [11, 12]. Moreover, it can cause heightened stress and anxiety for those living with CHB, and unnecessary lifestyle restrictions associated with fears of spreading HBV to others [9, 12, 13].

Previous studies have measured HBV knowledge among Asian Americans [6, 10, 13, 14], but few have explored HBV knowledge among a subset of Korean-American CHB patients who receive specialized hepatology care from a Korean clinician. Cultural concordance in care is thought to remove specific barriers, such as language, that Asian American patients would face otherwise [15]. By having a healthcare provider who speaks the same language and has the same cultural background, communication between patients and providers may be more effective, potentially leading to improved understanding of CHB among patients [15]. The objectives of this study are to assess the current state of HBV transmission knowledge among Korean-American CHB patients and to see whether language preference impacts their knowledge of Hepatitis B transmission.

## Methods

### Study Design and Participant Sample

This is an explanatory mixed-methods study using survey data and in-depth interviews [16]. This analysis is part of a larger prospective longitudinal cohort study that aims to look at disparities in liver disease progression among Korean Americans with CHB. Patients were recruited from two clinical sites in Philadelphia and Los Angeles, where they receive care from bilingual Korean-American hepatologists. The cohort enrollment criteria include individuals who are over 18 years old, have the cognitive ability to provide informed consent and complete study procedures, have been a recipient of care from the participating clinical site since at least 2016, and have a diagnosis of CHB, but without a prior diagnosis of hepatocellular cancer, or co-infection with hepatitis C or HIV. Recruitment occurred between August 2021 and January 2023. Participants could complete the survey and interview in Korean or English. More information about the study design for the overall study can be found here [17].

Medical record review identified 552 eligible patients, of whom 140 (25.4%) did not return for care nor respond to multiple contact attempts. Of 412 patients contacted, 4 (1%) declined to return to the clinic during enrollment, and 16 (3.8%) were ineligible after further screening. Of 392 eligible patients, 27 (6.8%) refused to participate, and 2 did not complete the enrollment questionnaire; therefore, the final analytic cohort includes 363 patients.

### Baseline Survey Measures

#### Outcome Measures

Transmission knowledge was measured with our previously validated index listing ten potential transmission modes with response choices of yes, no, or don’t know [10]. Each item was recoded (1 = correct, 0 = incorrect/don’t know), and summed scores of 0–10 were used to measure respondents’ knowledge, with higher scores indicating greater knowledge about HBV transmission. The summed score showed good reliability (alpha = 0.87), suggesting that the individual items reliably captured underlying knowledge of HBV transmission [18].

#### Independent Measures

Sociodemographic variables used in these analyses included respondent gender, age, country of birth, education, survey language preference, use of five possible sources for HBV-related information, having a primary care provider (PCP), years since HBV diagnosis, event precipitating HBV testing and diagnosis and awareness of infected family members.

### Qualitative Interviews

Approximately one year after enrollment, qualitative in-depth interviews were conducted with a subsample of participants. These interviews explored baseline findings to provide contextual understanding of participants’ lived experience with CHB. Participants were selected using a quota-sampling strategy to balance gender, geographic location, and language, and purposive sampling to capture cohort diversity in age, socioeconomic background, immigration stories, and health histories.

A sample size of 30 participants was selected a priori, based on the focused nature of the interview topic and the potential for richly descriptive interview content from each participant. Of the 363 participants in the total cohort, 49 participants were contacted sequentially as potential interview participants. Among the 49 contacted participants, 7 declined, 5 agreed but did not complete scheduling, and 7 did not respond despite repeated contact attempts.

Two bilingual research assistants conducted audio-recorded interviews over the phone in the participant’s preferred language. The interview guide used a phenomenological approach to explore each participant’s lived experience from diagnosis to the present. Participants provided verbal consent and received a $40 gift card.

Each interview was transcribed verbatim, and Korean interviews were translated into English. The first iteration of the codebook included deductive and inductive themes. Reliability was established by having four co-authors independently code two transcripts, adding additional themes and codes as needed, and discussing coding differences to reach consensus. Using the finalized codebook, two research assistants who conducted the interviews coded the remaining interviews, using NVivo14 for coding and thematic analysis.

### Analysis

All survey data were cleaned and analyzed using SPSS. Descriptive statistics were used to examine the sample’s baseline characteristics and distribution of responses to HBV knowledge questions. Bivariate associations were explored between the total HBV knowledge score and all independent variables. For categorical variables, mean group scores were compared using t-tests and p-values. For continuous variables, correlations between the HBV knowledge score and each variable were presented with p-values. A final multivariable linear regression was selected using a stepwise backward selection process. Thematic analysis of participants’ experience talking about HBV transmission with others was conducted for all qualitative in-depth interviews. Central themes with example quotes from participants are presented.

## Results

### Quantitative Findings

#### Table 1: Cohort Demographics

The cohort was 56% male, with an average age of 60 years, and an age range from 19 to 84 years. Most participants were first-generation (97%), college-educated (56%), preferred completing the survey in Korean (82%), and had a PCP (94%). Patients who preferred English were significantly younger, more likely to be born in the US, and more educated than patients who preferred Korean. Patients received their HBV diagnosis on average 27 years ago, 45% of participants were diagnosed through a regular checkup, and 67% knew that a family member also had HBV infection. Doctors and medical caregivers, newspapers and magazines, and the internet were the most common sources of information used to learn about HBV. Approximately 27% of participants used three sources of information to get HBV information, and 5% did not use any sources of information.

**Table 1:** Cohort characteristics (*N* = 363)

Characteristics	Total (*N* = 363)	English (*N* = 65)	Korean (*N* = 298)	*P*-value
Male	202 (55.6%)	42 (64.6%)	160 (53.7%)	0.108
Mean age *±* SD	60.1 *±* 10.7	49.3 *±* 11.0	62.5 *±* 9.0	< 0.001
Generation				
1st generation 2nd generation	352 (97.0%) 11 (3.0%)	54 (83.1%) 11 (16.9%)	298 (100.0%) 0 (0.0%)	< 0.001
Education level				
Less than high school High school graduate or GED Business or vocational school Some college College graduate Grad or professional school	23 (6.3%) 74 (20.4%) 6 (1.7%) 56 (15.4%) 133 (36.6%) 70 (19.3%)	0 (0.0%) 0 (0.0%) 2 (3.1%) 8 (12.3%) 26 (40.0%) 29 (44.6%)	23 (7.7%) 74 (24.8%) 4 (1.3%) 48 (16.1%) 107 (35.9%) 41 (13.8%)	< 0.001
Source of HBV information^1^				
Newspaper and magazine Family and friends Internet Special classes or events Talking to my doctor or people taking care of me	230 (63.4%) 139 (38.3%) 218 (60.1%) 40 (11.0%) 303 (83.5%)	29 (44.6%) 25 (38.5%) 39 (60.0%) 2 (3.1%) 54 (83.1%)	201 (67.4%) 114 (38.3%) 179 (60.1%) 38 (12.8%) 249 (83.6%)	< 0.001 0.847 0.809 0.027 0.711
Total number of information sources used				
0 1 2 3 4 5	19 (5.3%) 80 (22.2%) 62 (17.2%) 96 (26.7%) 76 (21.1%) 27 (7.5%)	4 (6.2%) 15 (23.1%) 13 (20.0%) 18 (27.7%) 11 (16.9%) 2 (3.1%)	15 (5.0%) 65 (21.8%) 49 (16.4%) 78 (26.2%) 65 (21.8%) 25 (8.4%)	0.660
Mean knowledge score	6.3 *±* 2.1	6.8 *±* 1.9	6.2 *±* 2.2	0.033
Has a PCP	342 (94.2%)	54 (83.1%)	288 (96.6%)	< 0.001
Mean years since HBV diagnosis	27.0 *±* 10.3	23.4 *±* 9.5	27.8 *±* 10.4	0.002
How did they get diagnosed				
Regular checkup Family history Pregnancy screening Presence of symptoms Blood donor screening Other	162 (44.6%) 59 (16.3%) 29 (8.0%) 28 (7.7%) 41 (11.3%) 44 (12.1%)	18 (27.7%) 16 (24.6%) 3 (4.6%) 7 (10.8%) 7 (10.8%) 14 (21.5%)	144 48.3%) 43 (14.4%) 26 (8.7%) 21 (7.0%) 34 (11.4%) 30 (10.1%)	0.006
Known family history of HBV	243 (66.9%)	48 (73.8%)	195 (65.4%)	0.192

*Note*. ^1^ Multiple responses are possible

#### Table 2: Knowledge Question Responses

The average knowledge score among all participants was 6.3 out of 10. Looking at each question of the knowledge scale, only 30% of all participants correctly identified that HBV is not transmitted through pre-chewed food or breastfeeding, and only 61% correctly answered that HBV could be transmitted through sexual intercourse. Most participants correctly identified that HBV cannot be transmitted through holding hands (90%) and that transmission could occur perinatally (82%). However, for any given question, 8.3–20.1% of the entire cohort responded “don’t know.” There were significant differences in responses by language for six transmission questions: sharing a toothbrush, eating pre-chewed food from an infected person, being coughed/sneezed on, sexual intercourse, holding hands, and breastfeeding. English-language participants were more likely to correctly answer that HBV could not be transmitted by eating pre-chewed food (46.2% versus 26.8%, *p* = 0.003), being coughed or sneezed on (78.5% versus 61.7%, *p* = 0.033), holding hands (98.5% versus 87.6%, *p* = 0.034), and a better understanding that HBV could be transmitted through sexual intercourse (76.9% versus 57.0%, *p* = 0.004). Korean-language participants were better able to identify that HBV can be transmitted by sharing a toothbrush (66.8% versus 47.7%, *p* = 0.012). Half of the Korean-language participants incorrectly responded that HBV could be transmitted through breastfeeding, while 32% of English-language participants did not know the answer.

**Table 2:** Transmission question results, by language preference (*N* = 363)

Transmission question	Total (*N* = 363)			English (*N* = 65)			Korean (*N* = 298)			*p*-value
	Yes	No	Don’t Know	Yes	No	Don’t Know	Yes	No	Don’t Know	
Mother to child during childbirth (Y)	**297 (81.8%)**	30 (8.3%)	36 (9.9%)	**51 (78.5%)**	7 (10.8%)	7 (10.8%)	**246 (82.6%)**	23 (7.7%)	29 (9.7%)	0.681
Food prepared by an infected person (N)	51 (14.0%)	**277 (76.3%)**	35 (9.6%)	6 (9.2%)	**52 (80.0%)**	7 (10.8%)	45 (15.1%)	**225 (75.5%)**	28 (9.4%)	0.460
Sharing a toothbrush (Y)	**230 (63.4%)**	88 (24.2%)	45 (12.4%)	**31 (47.7%)**	21 (32.3%)	13 (20.0%)	**199 (66.8%)**	67 (22.5%)	32 (10.7%)	0.012
Sharing food (N)	87 (24.0%)	**240 (66.1%)**	36 (9.9%)	10 (15.4%)	**47 (72.3%)**	8 (12.3%)	77 (25.8%)	**193 (64.8%)**	28 (9.4%)	0.187
Sharing a razor (Y)	**255 (70.2%)**	62 (17.1%)	46 (12.7%)	**48 (73.8%)**	7 (10.8%)	10 (15.4%)	**207 (69.5%)**	55 (18.5%)	36 (12.1%)	0.293
Eating food that has been pre-chewed by an infected person (N)	200 (55.1%)	**110 (30.3%)**	53 (14.6%)	24 (36.9%)	**30 (46.2%)**	11 (16.9%)	176 (59.1%)	**80 (26.8%)**	42 (14.1%)	0.003
Being coughed/sneezed on (N)	74 (20.4%)	**235 (64.7%)**	54 (14.9%)	7 (10.8%)	**51 (78.5%)**	7 (10.8%)	67 (22.5%)	**184 (61.7%)**	47 (15.8%)	0.033
Sexual intercourse (Y)	**220 (60.6%)**	97 (26.7%)	46 (12.7%)	**50 (76.9%)**	7 (10.8%)	8 (12.3%)	**170 (57.0%)**	90 (30.2%)	38 (12.8%)	0.004
Holding hands (N)	8 (2.2%)	**325 (89.5%)**	30 (8.3%)	0 (0.0%)	**64 (98.5%)**	1 (1.5%)	8 (2.7%)	**261 (87.6%)**	29 (9.7%)	0.034
Breastfeeding (N)	179 (49.3%)	**111 (30.6%)**	73 (20.1%)	23 (35.4%)	**21 (32.3%)**	21 (32.3%)	156 (52.3%)	**90 (30.2%)**	52 (17.4%)	0.011

*Note*. The bolded cells represent the correct answer

#### Table 3: Bivariate Associations

**Fig. 1:**
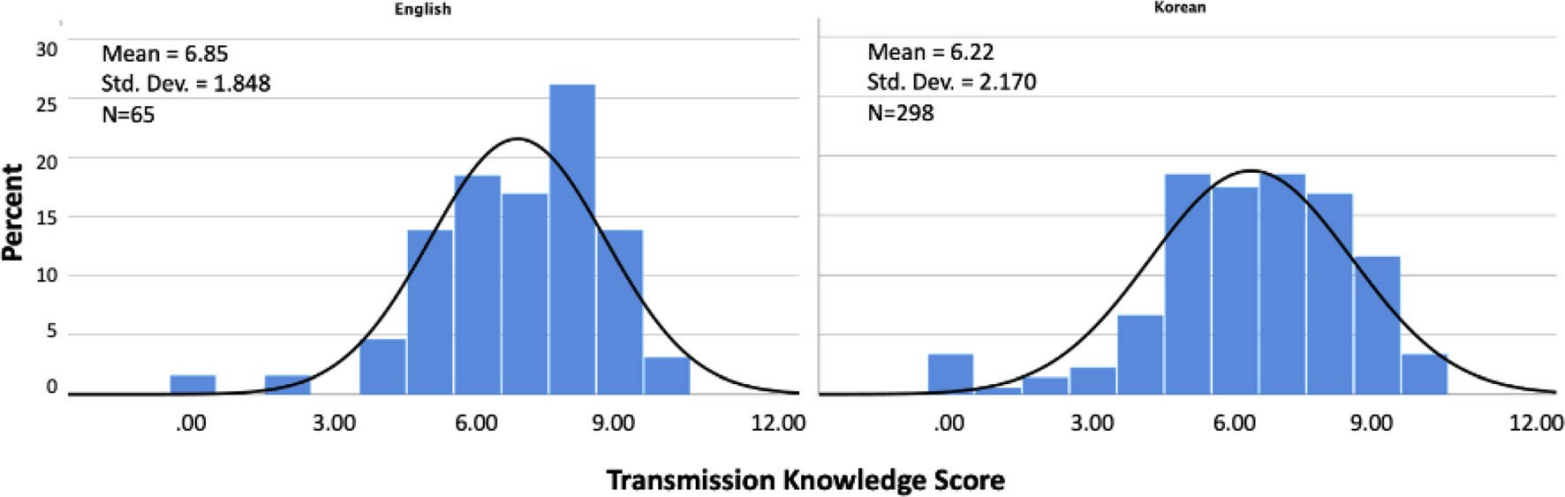
Total knowledge score by language preference (*N* = 363). Note. Range [0–10]; p-value = 0.033

Knowledge scores did not differ by gender, country of birth, or having a PCP. Between different languages, English-language participants scored significantly higher than Korean-language participants (6.9 versus 6.2, *p* = 0.033) (Fig. 1). Participants who used the internet to seek HBV information had significantly higher knowledge scores than those who did not (6.6 versus 5.9, *p* = 0.003). Those with a known family history of HBV had higher knowledge scores compared to those who did not (6.5 versus 5.9, *p* = 0.019). Older age was negatively correlated with knowledge score (-0.28, *p* < 0.001). Higher education level (0.16, *p* = 0.003) and number of information sources used (0.15, *p* = 0.005) were positively correlated with knowledge score.

**Table 3:** Bivariate associations and correlations between total knowledge score and cohort characteristics (*N* = 363)

Characteristics	Mean Total Knowledge Score	*P*-value
Gender		
Male Female	6.33 6.35	0.925
Generation		
1st generation 2nd generation	6.29 6.95	0.179
Preferred language		
Korean English	6.22 6.85	0.033
PCP		
Yes No	6.35 6.19	0.747
Information about HBV in newspapers and magazines		
Sometimes/Very often Never/Not often	6.50 6.08	0.075
Information about HBV from family and friends		
Sometimes/Very often Never/Not often	6.60 6.19	0.074
Information about HBV from the internet		
Sometimes/Very often Never/Not often	6.61 5.94	0.003
Information about HBV from special classes or events		
Sometimes/Very often Never/Not often	6.48 6.33	0.684
Information about HBV from talking to my doctor		
Sometimes/Very often Never/Not often	6.41 6.00	0.175
Known family history of HBV		
Yes No	6.54 5.93	0.019
	**Correlations**	**P-value**
Age	-0.28	< 0.001
Years since HBV diagnosis	0.04	0.414
Education	0.16	0.003
Total number of information sources used	0.15	0.005

#### Table 4: Multivariable Linear Regression

The final model contains three covariates that remained significantly associated with HBV transmission knowledge score: age, education level, and total number of information sources used. Older age remained negatively associated with knowledge score (β=-0.25, *p* < 0.001). More years of formal education (β = 0.09, *p* = 0.076) and a greater number of information sources used to seek HBV information (β = 0.12, *p* = 0.023) were positively associated with higher knowledge scores.

**Table 4:** Multivariable linear regression (*N* = 363)

	Unstandardized Coefficients	Standardized Coefficients	*P*-value
Age	-0.049	-0.250	< 0.001
Education	0.123	0.093	0.076
Total number of information sources used	0.178	0.116	0.023

### Qualitative Findings (Table 5)

The 30 in-depth interview participants were approximately balanced by gender (16 men/14 women), clinical site (15 Philadelphia/15 Los Angeles), and language (15 English/15 Korean). Average knowledge score among interview participants did not differ significantly from the overall cohort (7.0 versus 6.3, *p* = 0.09). A similar pattern is observed within language groups. Among all English participants, knowledge scores did not significantly differ between those who did and did not participate in the qualitative interview (7.4 versus 6.7, *p* = 0.189). Similarly, with Korean participants, knowledge scores did not significantly differ between those who did and did not participate in the qualitative interview (6.5 versus 6.2, *p* = 0.628).

**Table 5:** Qualitative quotes (*N* = 30)

**Doctor-Patient Communication**
**Addressing fears or misperceptions** * “Because my husband was running a bookstore in [name of city]. And there was this assistant, an employee there. And even though I’ve been a nurse. I don’t know everything 100%. So I thought that employee, the guy, might have been gay. So I wondered, “Was it because we shared the bathroom with him?” And when I asked [name of doctor] about it, [name of doctor] said that it’s not transmitted that way. It’s mostly transmitted through blood, serum, etc., not in that kind of situation.” [Korean-preference, F, 69]. * “When I had my son, my first kid, I was concerned about the transmission of it, but my OBGYN reassured me that because there’s vaccination now, as long as he’s vaccinated now, as soon as he’s delivered that he’s he would be fine and then they follow up with blood work to make sure you know, he’s ok. I was, I felt a little bit more comfortable after finding that out.” [English-preference, F, 41] * “Yes, that’s right. I had (hepatitis B) and I told the sanitation hospital about it. They told me that in my condition, I wouldn’t transmit it to others through food or anything, and as long as I didn’t exhaust myself, it was fine – I was just a carrier of hepatitis B.” [Korean-preference, F, 60].
**Application of knowledge** * “I understand quickly when the doctor explains it, but when I try to recall and use that knowledge later, I can’t.” [Korean-preference, M, 76]. * “I actually don’t think I really tried to understand. I believe that ‘it’s not about whether I understand or not, but rather that the doctor is an expert, and their judgment is correct.’ If the doctor says something needed to be done, I just did it.” [Korean-preference, F, 43] * “So anyway, regarding that, since childhood and when I was growing up, we didn’t really have much common sense about these things, right? After [name of doctor] informed me, I realized the connection and thought, “Oh, there’s this relationship, and this is what I need to do in such situations.’ Knowing beforehand is greatly beneficial in terms of prevention. I think prevention is really important.” [Korean-preference, M, 82] * “But [name of doctor] told me that I shouldn’t share toothpaste and toothbrushes. My oldest son is 52 years old, and we live in the same house, but we don’t share razors or anything. Now it’s become a habit, but I still am careful since my children have grown up.” [Korean-preference, M, 76] * “So, it was a long time ago, and at that time, there was a possibility of vertical infection like how I got it. So, the obstetrician told me that as soon as the baby is born, he should get an injection and that I should keep a close eye. And when I was preparing to give birth, the doctor told me that I should avoid breastfeeding just in case. Of course, I agreed not to do it.” [Korean-preference, F, 52]
**Misperceptions about transmission through shared food**
**General worry about sharing food** * “The doctor told me that I had to be careful with my relationships with others to prevent the spread, and especially, I was especially with food. That’s what the doctors advised me to be cautious about.” [Korean-preference, M, 82] * “So, when I go out to eat together, I don’t make a big deal about it or talk about it too much, but I am very mindful about certain things, like using my own utensils or serving myself side dishes first.” [Korean-preference, F, 52].
**Behavior restrictions despite having correct knowledge** * “When people around me have wrong ideas, I correct them. Especially when they worry about transmission and are reluctant to share meals. It had been a big problem at my workplace…. They think it is transmitted by sharing things like Korean stew or something. They think it can be transmitted that way… I really used to think that way myself, and there are so many people who still think that way…. Yes, but even after that, although I say I’m fine in my mind, I still try to reduce the change by trying to avoid sharing food as much as possible.” [Korean-preference, M, 47] * “Sure. I know that even though I carry hepatitis B, I can’t infect others because it’s not active. So, to be honest, it doesn’t affect me much. But when I’m in a place where a lot of people gather and we eat together, I do worry a bit. I find myself wondering, ‘Could I possibly transmit it to someone else?’ That kind of worry does exist. So, it’s not that it doesn’t affect me at all. I do have some concerns.” [Korean-preference, F, 52] * “For example, when I eat from my plate, I can share food with my daughter. I can offer her some. But people think that you can’t do that if you have Hepatitis B – that it’s something you shouldn’t do. But it’s not transmitted like that, right? But people think it’s a contagious behavior. So, I have no choice but to be careful. I do.” [Korean-preference, F, 62].
**Perpetuating misperceptions to others** * “Sometimes, Koreans are frustrating like that. It’s not a deadly disease, nor is it highly contagious. As long as we’re careful, like not sharing food or utensils, it’s fine. But people keep it a secret.” [Korean-preference, M, 76]. * “Yes, they all know. Why would I hide it? It’s not like it’s some contagious disease. But there are precautions to be taken, like when drinking alcohol, I always tell my friends not to share glasses. And I always say, ‘Don’t pass around the alcohol glasses when drinking.’ Now, people don’t do it as much. In the past, people used to freely give and receive drinks, but not anymore. Still, whenever there are those who still do, I make it a point to mention it. Don’t pass around alcohol glasses, and if food is served in an open manner, scoop out your portion and place it on your plate, and avoid clashing chopsticks.” [Korean-preference, M, 76]
**Reducing social interactions out of fear of transmitting to others** * “Yeah, if the articles that I read at the time was true. I could actually transmit through my saliva. So, I don’t wanna share my utensils or meals, anything like that. So, I started having less meals with friends or other people. And when I’m having a meal with other people at the same table, I want to make sure that I don’t dip my utensils to common plate.” [English-preference, M, 58] * “I mean there are things that the doctor tells you not to do. But in terms of going out and eating, sharing food or whatever. So those are the kind of things that I want to avoid.” [English-preference, M, 62] * “We need to be careful about what we eat and make sure not to inadvertently affect others. Since it can be transmitted, we need to be cautious and have to consider that. For instance, we shouldn’t share spoons or dishes, and if possible, it’s better not to eat together, that’s what I’ve been told.” [Korean-preference, M, 82]. * “[Name of doctor] advised, “In marital relations, you should do it this way, and if possible, it’s also good to use separate towels.” That’s what she told me. At first, I was careful, but then I just didn’t. We used the same towel, and it was like that. Overtime, I realized that transferring something through a towel isn’t that easy. So, Yes. Anyway. So, anyway, I just did it like that, and I don’t really meet many new people because of it.” [Korean-preference, F, 69]

#### Doctor-Patient Communication

Many participants referred to conversations they had with their healthcare providers, which had proven beneficial by helping them address fears or misperceptions about their condition. One woman recounts her experience with her OBGYN:

What I had my son, my first kid, I was concerned about the transmission of it, but my OBGYN reassured me…there’s vaccination now … I felt a little more comfortable after finding that out.

These conversations also educated patients about preventing further spread of infection:

But [name of doctor] told me that I shouldn’t share toothpaste and toothbrushes … we live in the same house, but we don’t share razors or anything. Now it’s become a habit, but I still am careful.

Although talking to providers was described as informative and beneficial, one patient has also expressed difficulty applying the knowledge they learned from their providers.

I understand quickly when the doctor explains it, but when I try to recall and use that knowledge later, I can’t.

Another patient expressed not feeling the need to comprehend the education they were receiving from their provider. Instead, they felt it was just a matter of adhering to their provider’s recommendation.

I actually don’t think I really tried to understand. I believe that ‘it’s not about whether I understand or not, but rather that the doctor is an expert, and their judgment is correct.’ If the doctor says something needed to be done, I just did it.

#### Misperceptions About Transmission Through Shared Food

Although HBV cannot be transmitted through saliva and sharing food, patients expressed general worry about eating with others.

The doctor told me that I had to be careful with my relationships with others to prevent the spread … especially with food.

There were also instances where patients knew that they could not spread HBV to others during shared meals but still felt the need to be cautious in front of others.

For example, when I eat from my plate, I can share food with my daughter …But people think that you can’t do that if you have hepatitis B – that it’s something you shouldn’t do … people think it’s a contagious behavior. So, I have no choice but to be careful.

Typically, beliefs held by others caused patients to be more cautious about their behavior, even if they knew it was unnecessary. In addition, some misinformed patients spoke about promoting excessive restrictions within their social circles.

It’s not like it’s some contagious disease. But there are precautions to be taken, like when drinking alcohol, I always tell my friends not to share glasses… and if food is served in an open manner, scoop out your portion and place it on your plate, and avoid clashing chopsticks.

At the most extreme, some participants have reduced social interactions out of fear of transmitting the virus to others.

Yeah, if the articles that I read at the time was true, I could actually transmit through my saliva. So, I don’t want to share my utensils or meals, anything like that. So, I started having less meals with friends or other people.

## Discussion

Health education about ways to reduce the transmission of HBV is important for patients to reduce the spread of the infection. Knowledge empowers individuals to have more agency to make informed decisions. Our findings show that knowledge is still inadequate, and opportunities exist to improve education in this population.

### Gaps in HBV Transmission Knowledge

The breakdown of knowledge score shows interesting patterns in gaps of knowledge in this population. Only 30% of the cohort knew that HBV could not be transmitted through breastfeeding or prechewed food from an infected person. Concerns may arise because HBV DNA can be detected in breast milk and the possibility of blood from cracked nipples [19]. If a nursing mother’s nipple is cracked and bleeding, the virus could be transmitted to the infant during nursing. However, this could only occur in the unlikely situation that the child also had an open wound which was exposed to the mother’s blood, leading to percutaneous transmission. Population-based studies in both the U.S. and China found that breastfeeding did not increase the risk of HBV transmission if the child received their first HBV vaccine at birth [20, 21]. Regardless of this information, providers may still be overly cautious of this risk and may not encourage HBV-infected mothers to breastfeed [22]. This is consistent with one woman’s account of her OBGYN’s recommendation against breastfeeding. However, breastfeeding provides many benefits to the infant and is encouraged for women with CHB [23, 24]. There are also concerns about prechewed food coming in contact with infected blood through a cut in the mouth. Transmission through this method is less documented for HBV, and few studies have shown clinical evidence to prove otherwise. Prechewing food for young children is a cultural practice not commonly practiced within Korean culture, so most of our participants may not be familiar with this.

There were also significant differences in understanding transmission routes between language groups. Sexual intercourse is one prominent route of transmission, but only 57% of Korean-language patients correctly answered that compared to 77% of English-language patients. A previous study of Korean American parents in New York found that only 23% of participants knew that HBV could be transmitted through sexual contact [6]. Although this was much lower than the current study population, one explanation may be attributed to differences in education regarding sexual health in Korea compared to the US. A survey conducted in Korea to assess the public’s general understanding of liver-related diseases found that approximately 60% of their sample did not know that HBV could be transmitted through sexual contact [25]. This is consistent with our results. Since our patients were diagnosed with HBV on average 27 years ago, many of them may have received their diagnoses while they were still in Korea. Results for our Korean language patients’ understanding of transmission through sexual intercourse may reflect cultural norms.

Sociodemographic factors contributing to health literacy may also contribute to higher scores among English-language participants. Health literacy is the ability to acquire, process, and understand basic health information [6, 26]. Although language was not significant in the final model, education attainment and the number of information sources used may have a more proximal impact on the pathway of effects. Suggesting that regardless of language, persons with CHB can acquire the knowledge they need if they can access information from multiple sources.

Regardless of language, many participants were unable to answer questions about HBV transmission. This is concerning as these patients have received specialized care for at least five years. The responses show a gap in health education and suggest an opportunity to improve knowledge about HBV transmission routes.

### Predictors of High HBV Transmission Knowledge Scores

It is also important to speculate as to why HBV transmission knowledge was lower among older respondents despite their tending to have more years of experience with the disease. This finding is consistent with other studies and may be explained in part by the life course trajectory of patients’ experiences of CHB as a chronic condition [27, 28].

Participants most commonly named “talking to my doctor” as their source of HBV information. Many patients would have received their initial CHB-related health education from their physician during diagnosis and treatment initiation. Although we do not know what information those clinicians provided, for older patients, whose diagnosis occurred decades ago, earlier information may now be outdated. Furthermore, as time progresses, the focus of clinical visits may shift to treatment and maintenance. Older patients may not be able to recall the health education they previously received, and clinicians may not see it as relevant to re-educate older patients about transmission via routes such as breastfeeding or intercourse.

Print media and the Internet were other sources of information used. This reflects other passive and active ways patients can acquire HBV knowledge outside their regular medical appointments. It suggests that having multiple points of opportunity for patients to learn about HBV throughout their disease journey can increase their knowledge about transmission routes.

### Provider Influence on HBV Transmission Knowledge

Language and cultural differences are barriers that can hinder medical care for CHB among Asian American populations [29]. Asian American patients who felt that their doctor did not understand their culture and values were more likely to report dissatisfaction with their quality of care and less likely to trust their clinician [15]. All patients in this study received specialized liver care from Korean-American hepatologists fluent in English and Korean. This model of ethnically concordant care reduces barriers by making communication and sharing information easier. Participants also shared in their qualitative interviews how their hepatologist and OBGYN were influential in teaching them about HBV transmission.

Our results underscore the importance of healthcare providers as trusted sources of information and emotional support. However, qualitative interviews revealed concerns about patients’ comprehension and application of the information they receive. Culturally, physicians, especially specialists, are highly respected and are perceived to have absolute authority over a patient’s treatment plan [30]. This hierarchical relationship may make it difficult for patients to inquire freely and not appear rude to an authority Figs. [30, 31]. Although patient adherence is a positive attribute, it is also important for patients to feel agency in comprehending medical advice given by their clinicians. Unless they can comprehend it, they are unlikely to be able to recall any knowledge in the future and apply it.

### Misinformation and Stigma among CHB Patients

Our qualitative results show that misperceptions about spreading HBV through food remain prevalent in this population. In Korean culture, sharing food, such as soups and stews, during meals is very common. Participants mentioned avoiding sharing food and utensils to reduce HBV transmission, consistent with other studies of Asian Americans with similar eating practices [6, 14, 32]. In our analysis, some participants believe HBV can spread through saliva and sharing food, leading to social restrictions. Others understand transmission routes but avoid sharing to prevent stigma. HBV-related stigma is a persistent issue that can cause fear, anxiety, and worry among CHB patients, and impact health-seeking behaviors and disease management [33–36]. Lack of education and misinformation about HBV transmission is a driver of HBV-related stigma [37–39]. Proactively educating the general population is important in reducing misinformation and will help foster more positive environments for CHB patients.

### Strengths and Limitations

This study utilizes a sequential mixed-methods design using data from structured surveys and in-depth interviews. This design allows for a more comprehensive understanding of knowledge in this population of CHB patients [16]. A strength of this study is that the population was specific to Korean-American CHB patients receiving care from Korean hepatologists. Previous studies about HBV among Korean-Americans have been limited to those in the community or those receiving care in a care system [6, 32, 40]. Prior studies have not looked at the effects of racial concordance of care in this population. Findings from this study provide insight into existing barriers to knowledge within this care model.

One limitation is that the survey was cross-sectional, so we must be cautious when considering associations between knowledge scores and independent predictors. For example, it is possible that greater knowledge of HBV could motivate a patient to seek additional sources of information, that these relationships are bidirectional, and that the association between older age and less knowledge is both an aging and a cohort effect. The interviews partially mediate this limitation by allowing patients to recount their life managing CHB and offer insight into their lived experiences. A second limitation is that our survey responses were all self-reported. Patients diagnosed decades ago may have difficulty responding to questions that require them to think back to earlier parts of their disease journey. Finally, we did not ask direct questions about transmission knowledge in the qualitative interviews. However, many participants did share about their knowledge acquisition experience and strategies they used to manage HBV.

## Conclusions

These results suggest that despite receiving culturally concordant care from expert hepatologists, some Korean-American CHB patients have an inadequate understanding of transmission. Misperceptions about transmission pathways still exist, and older age, less formal education, and fewer sources of HBV information may result in lower knowledge. Continued health education after the point of diagnosis is important in maintaining accurate knowledge and reducing misperceptions about HBV transmission. Although there is no clinical risk associated with patients being cautious about the spread of HBV, being overly cautious can negatively impact the patient’s quality of life as they navigate the already challenging task of managing a chronic condition. Results from this study and existing literature also highlight the role of the provider as a trusted source of information in providing health education and emotional support. Further research should be done to identify additional factors that influence knowledge acquisition and retention. Doing so will be important for developing culturally appropriate health education for this population.
